# Testing Real-World Application of Appropriateness Criteria of Single Photon Emission Computed Tomography (SPECT) In Two Egyptian Hospitals

**DOI:** 10.1186/s43044-019-0022-2

**Published:** 2020-01-10

**Authors:** Adham Abdeltawab, Hazem Mansour, Mona Rayan

**Affiliations:** Cardiology Department, Ain Shams University Hospitals, Faculty of Medicine, Ain Shams University, Abbassia, Cairo P.O. 11381 Egypt

**Keywords:** SPECT, MPI, stress, Coronary artery disease, viability test

## Abstract

**Background:**

The American College of Cardiology pioneered appropriateness criteria for single-photon emission computed tomography myocardial perfusion imaging in 2005 to account for evidence-based clinical relevance of stress perfusion imaging.

Our aim was to assess and compare appropriateness use criteria in in Kobry al Kobba military hospital and Ain Shams University hospitals.

**Methods:**

All patients were subjected to thorough history taking, calculation of pretest probability and Framingham risk score, determination of appropriateness use criteria and stress-rest Tc 99m imaging to detect the presence of ischemia and one day Tc 99m imaging to detect viability.

**Results:**

The study included 442 patients with mean age of 56.5 years, with male predominance (77%), 38% were diabetics and 58% had hypertension.

Seventy-eight percent of patients had appropriate tests, uncertain tests in 12% and 10% inappropriate studies. 47% of appropriate tests show positive results of SPECT.

**Conclusion:**

We concluded that appropriateness criteria are effective in identifying appropriateness of SPECT in a diverse patient population.

## Background

There has recently been an explosive growth in cardiovascular imaging, with stress testing demonstrating 6.1% annual increase versus 2% for cardiac catheterization, 0.8% for percutaneous intervention, and 0.1% for acute myocardial infarctions in population-based study of the United States Medicare patients [1] [2].

The American College of Cardiology pioneered appropriateness criteria for single-photon emission computed tomography (SPECT) myocardial perfusion imaging (MPI) in 2005 [1]. The criteria were developed to account for evidence-based clinical relevance of stress perfusion imaging and were the first cardiology specific document to address appropriateness. The criteria relied on the modified RAND/UCLA methodology to identify 52 common clinical scenarios and was divided into appropriate, uncertain, and inappropriate indications [1, 3].

New designations were suggested for some indications when SPECT criteria were reviewed in 2007 [4]. An update on appropriateness criteria was published in May 2009 [5].

Despite these developments, the clinical use of the appropriateness criteria has not become standard of practice for physicians [6]. Overall, approximately 64% of stress studies are appropriate. Approximately 14% of SPECT and 18% of stress echocardiography studies are performed for inappropriate indications, and approximately 10% of all patients are unclassifiable [6].

In a prospective multicenter trial, the appropriateness criteria were evaluated in 7 physician practices of various size in partnership with United HealthCare. Overall, 14% of all studies were inappropriate, most in asymptomatic individuals [5].

### Aim of the work

Was to assess and compare appropriateness use criteria and the downstream use of resources as defined by patient outcomes in two different nuclear centers in Cairo.

## Methods

### Patient Population

#### Inclusion criteria

All patients older than 18 years undergoing SPECT in Kobry al Kobba military hospital and Ain Shams University hospitals and capable of primary decision making were included throughout the period from February 2012 to august 2012.

#### Exclusion criteria

1. Chronic illnesses or malignancy with estimated life expectancy of less than 1 year,
2. Unwilling to give informed consent,
3. Not accepting record access and phone call follow up.

The final cohort consisted of 442 patients.

### Methods

After having a verbal consent of including the patients’ data in the study, the following were recorded:

### History taking:

1. Age in years.
2. Gender.
3. Cardiovascular risk factors: arterial hypertension, diabetes mellitus, smoking and positive family history of premature cardiovascular diseases.
4. Known history of ischemic heart disease (defined as prior myocardial infarction demonstrated whether by ECG changes or revascularization history).

**Pretest Probability of CAD**: was determined for Symptomatic patients as in Table 1

**Fig. 1:**
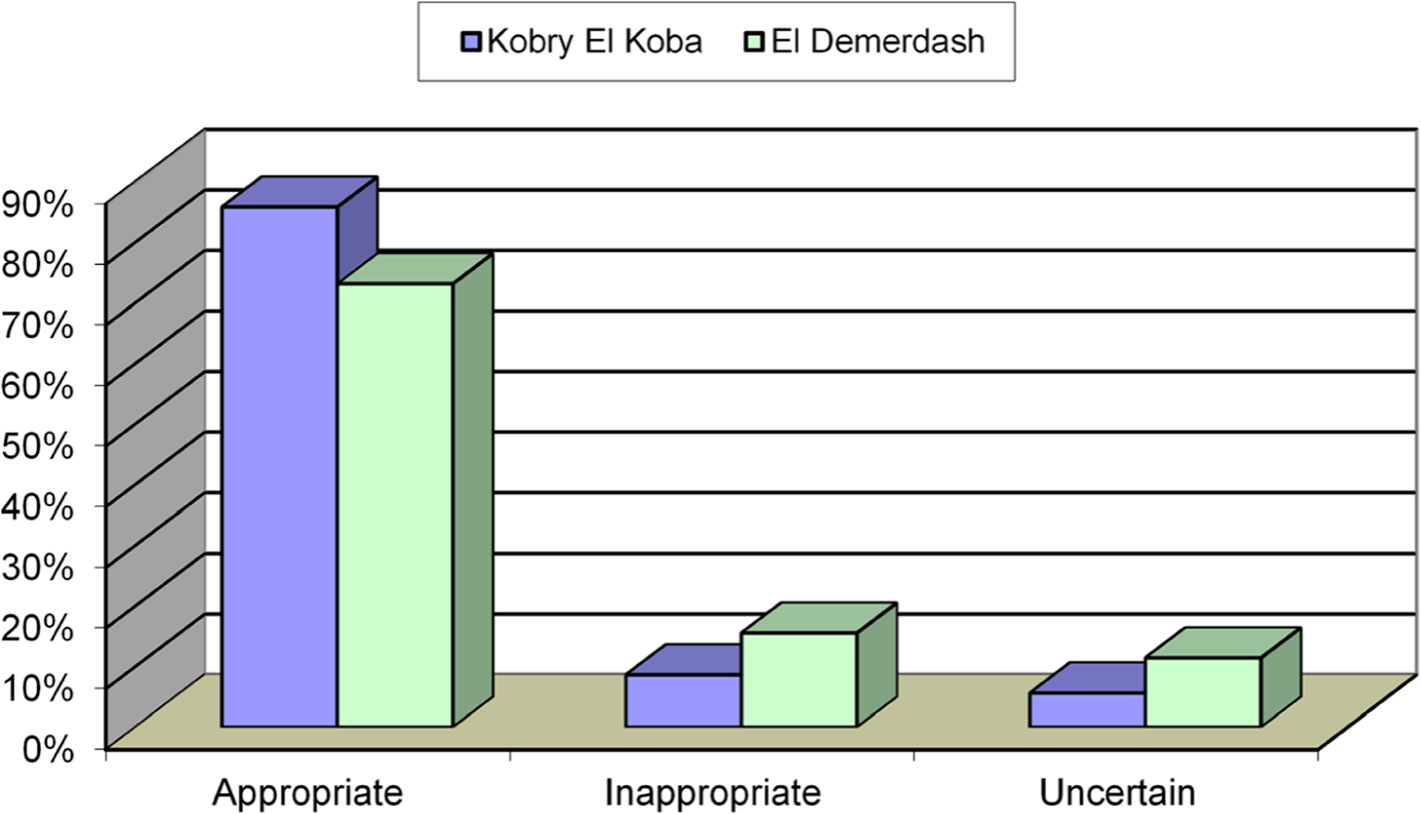
appropriateness criteria in both groups

**Table 1:**
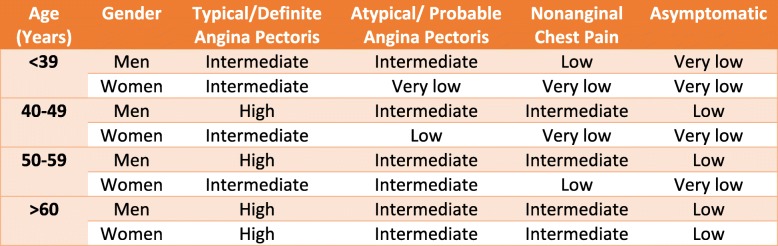
Pretest probability of CAD according to age, gender and chest pain

With High meaning greater than 90% pretest probability. Intermediate: Between 10% and 90% pretest probability, low: Between 5% and 10% pretest probability, and very low: Less than 5% pretest probability. Modified from the ACC/AHA Exercise Testing Guidelines to reflect all age ranges [7].

**The Framingham Risk Score (FRS)**: was done for patients with no symptoms and no history of ischemic heart disease as shown in Table 2

**Table 2:**
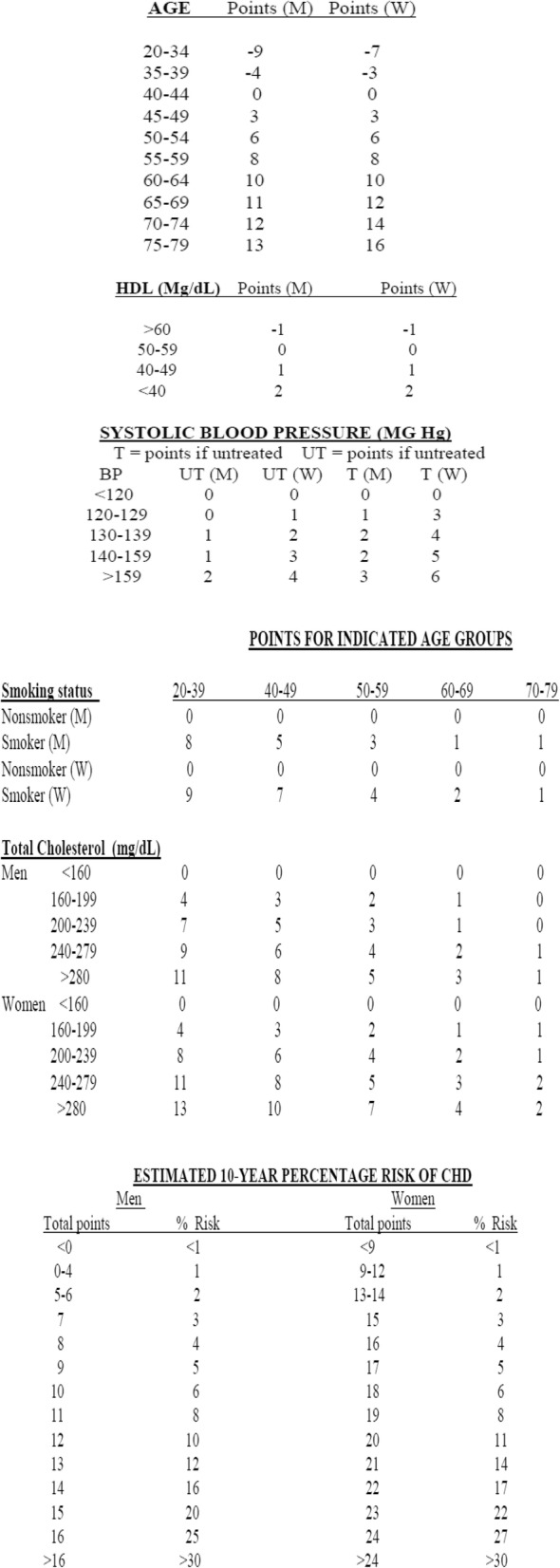
Framingham Risk Score Calculation

**Pre-procedural ECGs were analyzed for:**

- Presence of left bundle branch block.
- Healed infarction: e.g. Q waves in the ECG.
- Atrial fibrillation.
- Non-specific ST and T wave changes.

### Appropriateness use criteria: see Additional file 1

Appropriateness was determined prospectively based on symptoms, pre-test likelihood of disease [8], and Framingham risk scores, FRS [9].

### Single photon emission computed tomography

- **Stress-rest gated Tc99m Sestamibi scan:**

To detect the presence, severity and distribution of ischemia. For all stress imaging, the mode of stress testing was exercise for patients able to exercise.

For patients unable to exercise, pharmacologic stress testing was used. Further background on the rationale for the assumption of exercise is available in the ACC/AHA 2002 guideline Update for Exercise Testing [7].

### Protocol

**A 2day protocol** was used (day 1 for stress image and day 2 for rest one) by obtaining:

- **Stress imaging:**

After fasting for at least 4 hours and abstinence from nitrates, beta blockers and calcium channel blockers; images were taken approximately 45-60 min after injection of 22-30 mCi of technetium99m Sestamibi through an indwelling intravenous cannula.

#### Mode of stress

1. Exercise stress testing: the radiotracer was injected at peak exertion at target heart rate (85% of age predicted maximum heart rate), proper analysis of stress data was done as follows:

- Protocol used.
- Duration of exercise.
- Percent of heart rate achieved.
- Blood pressure response.
- Cause of termination: Occurrence of chest pain or significant stress induced ECG changes.

2. Pharmacological stress test: indications:

- Inability to exercise.
- Left bundle branch block.
- Contraindication to exercise stress test e.g. severe bronchial asthma.

**Pharmacological stress** was done using Dipyridamole (infusion rate of 0.5 gm/kg/4 min) or Dobutamine (infusion rate of 10 to 40 ug/kg/min) and the radiotracer was injected at maximum dose or target heart rate.

b. **Rest images:**

Obtained 1day after stress image, approximately 1-1.5 hours after injection of 22-30 mCi of technetium 99m Sestamibi.

- **One day rest imaging:** was done to assess viability after the injection of 70 mg of Trimetazidine 1 hour before injection of the radiotracer.

All radionuclide perfusion imaging indications used electrocardiogram (ECG) gating, whenever possible, with the determination of global ventricular function (i.e., left ventricular ejection fraction) and regional wall motion as part of the evaluation.

In the setting of a known acute coronary syndrome (ACS), the use of stress testing was performed in conjunction with pharmacologic stress testing, not exercise.

### Image acquisition and reconstruction

Tomographic images were obtained using a rotatory dual head cardiac dedicated gamma camera (cardio MD, Phillips medical equipment’s) in Ain Shams University hospitals and (dual head symbia II, Simens) in Kobry Al Kobba hospital with a low energy, general purpose collimator interfaced to a computer.

An arc of 180 degree was used, spanning from the 45 degree right anterior oblique projection to the 45 degrees left posterior projection.

### Image interpretation

Basal myocardial perfusion was assessed using the semiquantitative analysis on the 17-segment scoring system, as standardized by the cardiac imaging committee of the council on clinical cardiology of the American Heart Association [10].

### Nomenclature and location of the myocardial segments

#### Perfusion analysis was translated into

a- **Segmental perfusion score:** Perfusion was graded within each segment on a scale of 0 to 4, with 0 representing normal perfusion and 4 representing a very severe perfusion defect.
b- **The summed stress score (SSS):** which was the sum of the 17 segmental scores from the stress images representing the extent and severity of stress perfusion abnormality, the magnitude of perfusion defects related to both ischemia and infarction, as shown in Table 3 [11].
c- The summed rest score (SRS): which was the sum of the 17 segmental scores from the rest images representing the extent of infarction.
d- The summed difference score (SDS): which was derived by subtracting SRS from the SSS and represents the extent and severity of stress – induced ischemia. The digitalized images were evaluated by an experienced reader who was unaware of the clinical outcome and ECG findings of the patients.

**Table 3:** Summed stress score according to cedars-sinai scoring system. Other centers may use alternative scoring systems with varying cutoffs [11]

Summed stress score	Indication
< 4	Normal
4-8	Mildly abnormal
9-13	Moderately abnormal
> 13	Severely abnormal

### SDS score category [11]

0-1 Normal

2-4 Mild ischemia

5-7 Moderate ischemia

> 7 Severe ischemia

### Data management

- Data was analyzed on an IBM personal computer, using statistical package for special science (SPSS) software computer program version 15.
- Data was described using mean ± standard deviation (SD) and frequencies according if they were quantitative or qualitative respectively.
- **Independent student T test** was used for comparing of quantitative variables between two groups. **Chi square test** was used for comparing of distribution of qualitative variables between different groups.
- The significance of the results was assessed in the form of
  P value differentiated into:
- Non-significant: when *P* value > 0.05
- Significant: When *P* value < 0.05
- Highly significant: When *P* value < 0.001.

## Results

In the present study, 442 patients derived from two territory hospitals in Cairo (Ain Shams university hospital and Kobry El-Kobba military hospital).

The data of these patients were used to apply for the appropriateness criteria of performing myocardial perfusion scan in the two centers.

Based on the referral center results were divided into two groups:

- **Group I:** it included 245 patients derived from Ain Shams nuclear cardiology lab.
- **Group II:** it included 197 patients recruited from Kobry El-Kobba nuclear lab.

### Demographic data

There were 340 males (77%) and 102 females (23%) with a mean age of 56.5 +-9.8 years. Males were dominant in the group derived from the military hospital compared to patients derived from Ain Sams university hospital. Table 4, Fig. 1.

**Table 4:** Age and gender distribution of both groups

		Group I N=245		Group II N=197		Chi-square test
		No.	%	No.	%	P-value
Gender	Female	91	37.10%	11	5.60%	0.000
	Male	154	62.90%	186	94.40%	
Age	Mean ±SD	56.45 ± 9.47		56.49 ± 9.80		0.966

### Risk factors

Shown in Table 5.

**Table 5:** Risk factor distribution among both groups

	Group I *N*=245		Group II *N*=197		Chi-square test
	No.	%	No.	%	*P*-value
Smoking	67	27.30%	59	29.90%	0.547
Hypertensive	141	57.60%	115	58.40%	0.861
Diabetic	92	37.70%	76	38.60%	0.851

### Indications to undergo the test

Screening for CAD was the indication in 286 patients (65%), post MI angina was the indication in 80 patients (18%), post PCI angina was found in 117 patients (26%) and in 29 patients (6%) post CABG angina was the cause of referral.

Viability assessment was the referring cause in 31 patients (7%).

As regards the cause of referral there was significant difference between results in both groups as shown in Table 6.

**Table 6:** Indications of SPECT in both groups

	Group I *N*=245		Group II *N*=197		Chi-square
	No.	%	No.	%	*P* value
Screening for CAD	130	53%	159	79%	0.000
Infarction	35	14%	45	23%	0.02
PCI	64	21.60%	53	32.50%	0.01
Viability	26	11%	5	3%	0.001
CABG	8	3.30%	21	10.70%	0.002

### Pretest probability

By applying pretest probability, there were 296 patients with high or intermediate probabilities (67 %) and 146 patients with low probability for IHD (33%) in the whole study group.

In group I, the low pretest probability was significantly higher than group II, whereas the high or intermediate probability was significantly higher than group I. Table 7.

**Table 7:** Pretest probability in both groups

	Group I *N*=245	Group II *N*=197	*P* value
High or intermediate pretest probability	141 (58%)	155 (79%)	0.000
Low pretest probability	104 (42%)	42 (21%)	0.000

### Framingham Risk Score (FRS)

After excluding patients with documented CAD, there were 54 patients eligible for FRS (42 patients in group I and 12 patients in group II)

High and intermediate risk profile were significantly higher in group II s compared to group I. Table 8.

**Table 8:** Distribution of different categories according to FRS in both groups

	Group I N=245	Group II N=197	*P* value
Very low and low	25 (60%)	6 (50%)	0.005
Intermediate	5 (12%)	2 (17%)	
High	12 (28%)	4 (33%)	

**Pre-procedural ECG:** shown in Table 9.

**Table 9:** Pre-procedural ECG findings in both groups

	Group I *N*=245	Group II *N*=197	*P* value
Normal *n*(%)	95 (39%)	41 (21%)	NS
Pathological Q waves	35 (14%)	45 (23%)	NS
Non-specific changes	94 (38%)	99 (50 %)	NS
AF	3 (1%)	2 (1%)	NS
LBBB	5 (2%)	6 (3%)	NS
RBBB	9 (4%)	8 (4%)	NS
IVCD	4 (2%)	-	

### Myocardial perfusion imaging

There was no significant difference between both groups as regards the mode of stress used and procedural findings; Table 10, 11,12.

**Table 10:** Mode of stress in both groups

	Group I *N*=245	Group II *N*=197	*P* value
Rest imaging *n*(%)	26 (11%)	5 (3%)	0.001
Treadmill stress *n*(%)	151 (62%)	141 (72%)	0.66
Pharmacological *n*(%)	68 (28%)	51 (26 %)	0.66

**Table 11:** Development of symptoms during exercise test in both groups

	Group I *N*=245	Group II *N*=197	*P* value
Chest pain *n*(%)	35 (16%)	75 (39%)	
Target heart rate *n*(%)	143 (94%)	111 (79%)

**Table 12:** Results of MPI in both groups

	Group I *N*=245	Group II *N*=197	*P* value
Positive scan *n*(%)	131 (53%)	120 (61%)	
Negative scan *n*(%)	114 (47%)	77 (39%)	

### Appropriateness use criteria

Appropriateness use criteria in both centers shown in Tables 13, 14, 15.

**Table 13:** Appropriateness criteria in both groups

AUC	Group I		Group II		Chi square
	No.	%	No.	%	*P*-value
Appropriate	179	73%	169	86 %	0.005
Inappropriate	38	16%	17	8 %	
Uncertain	28	11%	11	6 %

**Table 14:** The number and percentage of each appropriateness criteria in both groups

AUC	Group I		Group II	
	No.	%	No.	%
Detection of CAD, symptomatic with intermediate pretest probability, able to exercise.	32	13.1	9	4.6
Detection of CAD, symptomatic with intermediate pretest probability, unable to exercise.	9	3.7	3	1.5
Detection of CAD, symptomatic with high pretest probability.	52	21.2	36	18.3
Risk assessment, post revascularization.	19	7.8	67	34
Risk assessment, with known chronic stable angina and coronary stenosis.	31	12.7	48	24.4
Detection of CAD, asymptomatic, high FRS	8	3.3		
Viability	26	10.6	6	3
Evaluation of ventricular function, with use of cardiotoxic drugs	2	0.8		
Detection of CAD, symptomatic, with low or intermediate FRS.	35	14.3	10	5.1
Known CAD, prior test result with normal coronary angiography	2	0.8		
Detection of CAD, symptomatic with low pretest probability.			3	1.5
Post revascularization, asymptomatic.	1	0.4		
Risk assessment, known CAD.	3	1.2	2	1
Risk assessment, post revascularization.	25	10.2	9	4.6
Acute coronary syndrome, primary PCI, asymptomatic.			4	2

**Table 15:** The number and percentage of each appropriateness criteria in relation to results of MPI in both groups

		SPECT				Chi-square test	
		Negative		Positive		X^2^	*P*-value
		No.	%	No.	%		
Group I	Appropriate	77	67.50%	102	77.90%	14.479	0.001
	Inappropriate	28	24.60%	10	7.60%		
	Uncertain	9	7.90%	19	14.50%		
Group II	Appropriate	62	80.50%	107	89.20%	5.130	0.077
	Inappropriate	11	14.30%	6	5.00%		
	Uncertain	4	5.20%	7	5.80%		

## Discussion

The objective of the current study is to identify clinical value of appropriateness use criteria in various patient and physician groups and to focus on downstream use of resources in relation to appropriateness.

This study evaluated SPECT appropriateness criteria in a diverse patient population. It included all patients undergoing SPECT in Kobry al Kobba military hospital and Ain Shams University hospitals throughout the period from February 2012 to august 2012.

It was noted that men were predominantly higher than women with 77% versus 23% respectively. Furthermore, men were higher in the group of Kobry Al Kobba military hospital (94%) than patients of Ain Shams University (62%).

This is higher than the results found by Regina et al. who investigated 570 patients with 55% of men and 45 % of women [12].

Diabetes, hypertension and smoking were investigated in our study as risk factors for ischemic heart disease and we found that 28% of patients were smokers, 58% hypertensive patients and 38% diabetics.

This is compared to Raymond J. et al., who investigated 284 patients undergoing SPECT imaging and found that 48% of patients were smoking, 71% with hypertension and 27% with diabetes [6].

Most of our patients had high or intermediate pretest probability 66%. It was noted that more patients with high or intermediate pretest probability were found in Kobry Al Kobba military hospital (79%) versus (58%) in Ain Shams university hospital.

On the other hand Regina et al. 2011 found that 51% of patients had low pretest probability and only 6% had high pretest probability [12].

FRS was calculated for asymptomatic patients with no history of CAD which predict 10-year incidence of CAD, most of the patients are in the low risk groups (57%).

This is supported by Regina et al. who found that 51% of the patients fall in the low risk category [12].

In the present study, the overall percent of appropriate studies was 78%. This is within the range found by Gibbons et al. 2008 who investigated the performance of appropriateness criteria for stress SPECT in 284 patients and found that 64% of SPECT studies were appropriate.

Also, Regina et al. found overall appropriate studies in the range of 63% [12].

On the other hand, we found that the overall inappropriate studies were forming 10% of all the studies done.

This finding is within the range reported by Gibbons et al. and Regina et al. which was (14%) [6, 12].

In our work, the uncertain or unclassified studies were found to be 15% of all studies.

This is supported Gibbons et al. who found that 10% of his studies were unclassified, but our findings were higher than that reported by Regina et al. who reported a 3% of unclassified studies [6, 12].

As regards center related findings, it was notable that the uncertain and inappropriate studies were higher in the university nuclear lab than that done in the military nuclear lab (15% versus 7%) respectively. This may be explained by the inclusion of more females in the university.

This finding was explained by Gibbons et al. who reported that almost 50% of inappropriate test were asymptomatic patients with a low FRS who were referred for screening for CAD [6].

The second large group of inappropriate tests was done in patients under consideration of intermediate risk surgery who had good exercise capacity and no or minor risk factors, and the third group of inappropriate patients were symptomatic patients with low pretest probability, and the final group were patients for low risk non-cardiac surgery [6].

These findings prove that specific scenarios may differ from institution to institution and the recognition of these general patterns in appropriateness criteria should be helpful to clinicians.

Women were more symptomatic that men and had a lower prevalence of known CAD.

### Limitations

Smaller number of included patients derived from 2 centers only over a relatively short period.

The inherited defects of FRS and its applicability on different ethnic groups such as Egyptians.

## Conclusion

Appropriateness criteria are effective in identifying appropriateness of SPECT in a diverse patient population. More appropriate studies were observed in patients obtained from both sides of examination.

This study demonstrates the application of appropriateness criteria to attempt quality improvement in the clinical use of stress cardiac imaging.

### Recommendations

Other long-term studies that include larger number of patients.

We encourage others to perform similar studies in their own institutions, hospitals, and private practices. Such efforts are only a first step towards the ultimate goal of quality improvement in this area and thereby contribute to increased efficiency in our healthcare system.

## Data Availability

Tables summing up our data is included in the manuscript. The datasets used and/or analyzed during the current study are available from the corresponding author on reasonable request.

## Abbreviations

SPECT: Single-photon emission computed tomography
MPI: Myocardial perfusion imaging
ECG: Electrocardiogram
CAD: Coronary artery disease
FRS: The Framingham Risk Score
SSS: Summed stress score
SRS: Summed rest score
SDS: summed difference score
PCI: Percutaneous coronary intervention
CABG: Coronary artery bypass graft
